# Improving the second-tier classification of methylmalonic acidemia patients using a machine learning ensemble method

**DOI:** 10.1007/s12519-023-00788-6

**Published:** 2024-02-24

**Authors:** Zhi-Xing Zhu, Georgi Z. Genchev, Yan-Min Wang, Wei Ji, Yong-Yong Ren, Guo-Li Tian, Sira Sriswasdi, Hui Lu

**Affiliations:** 1 Shanghai Engineering Research Center for Big Data in Pediatric Precision Medicine, Center for Biomedical Informatics, Shanghai Children’s Hospital, School of Medicine, Shanghai Jiao Tong University, Shanghai, China; 2 Center of Excellence in Computational Molecular Biology, Faculty of Medicine, Chulalongkorn University, Bangkok, Thailand; 3 Newborn Screening Center, Shanghai Children’s Hospital, School of Medicine, Shanghai Jiao Tong University, Shanghai, China; 4 SJTU-Yale Joint Center for Biostatistics and Data Science, National Center for Translational Medicine, Shanghai Jiao Tong University, Shanghai, China; 5 Center for Artificial Intelligence in Medicine, Research Affairs, Faculty of Medicine, Chulalongkorn University, Bangkok, Thailand; 6 State Key Laboratory of Microbial Metabolism, Joint International Research Laboratory of Metabolic & Developmental Sciences, Department of Bioinformatics and Biostatistics, School of Life Sciences and Biotechnology, Shanghai Jiao Tong University, Shanghai, China

**Keywords:** Machine learning, Methylmalonic acidemia, Second-tier test, Stacking

## Abstract

**Introduction:**

Methylmalonic acidemia (MMA) is a disorder of autosomal recessive inheritance, with an estimated prevalence of 1:50,000. First-tier clinical diagnostic tests often return many false positives [five false positive (FP): one true positive (TP)]. In this work, our goal was to refine a classification model that can minimize the number of false positives, currently an unmet need in the upstream diagnostics of MMA.

**Methods:**

We developed machine learning multivariable screening models for MMA with utility as a secondary-tier tool for false positives reduction. We utilized mass spectrometry-based features consisting of 11 amino acids and 31 carnitines derived from dried blood samples of neonatal patients, followed by additional ratio feature construction. Feature selection strategies (selection by filter, recursive feature elimination, and learned vector quantization) were used to determine the input set for evaluating the performance of 14 classification models to identify a candidate model set for an ensemble model development.

**Results:**

Our work identified computational models that explore metabolic analytes to reduce the number of false positives without compromising sensitivity. The best results [area under the receiver operating characteristic curve (AUROC) of 97%, sensitivity of 92%, and specificity of 95%] were obtained utilizing an ensemble of the algorithms random forest, C5.0, sparse linear discriminant analysis, and autoencoder deep neural network stacked with the algorithm stochastic gradient boosting as the supervisor. The model achieved a good performance trade-off for a screening application with 6% false-positive rate (FPR) at 95% sensitivity, 35% FPR at 99% sensitivity, and 39% FPR at 100% sensitivity.

**Conclusions:**

The classification results and approach of this research can be utilized by clinicians globally, to improve the overall discovery of MMA in pediatric patients. The improved method, when adjusted to 100% precision, can be used to further inform the diagnostic process journey of MMA and help reduce the burden for patients and their families.

**Graphical Abstract:**

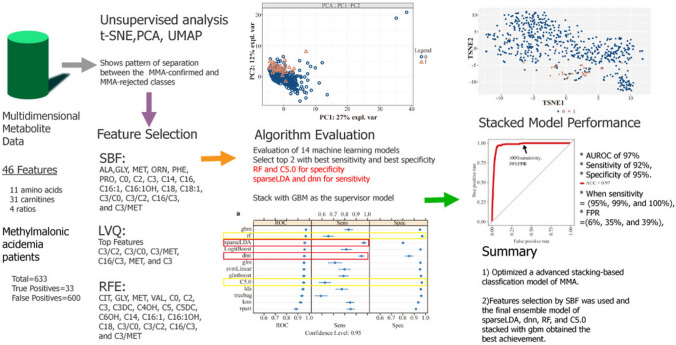

**Supplementary Information:**

The online version contains supplementary material available at 10.1007/s12519-023-00788-6.

## Introduction

Methylmalonic acidemia (also aciduria) (MMA) [international classification of diseases (ICD) 10: E71.1, Online Mendelian Inheritance in Man (OMIM) 251000, mut(0) type], first described as an inborn metabolism error in the year 1967 [1, 2], is a disorder with an autosomal recessive inheritance pattern, which occurs when an offspring inherits one copy of the mutated gene from each parent [3]. MMA is characterized by disrupted metabolism of certain amino acids, particularly the branched ones—isoleucine (ILE), leucine (LEU), and valine (VAL). It is a classical organic acidemia leading to the buildup of toxic levels of methylmalonic acid in the blood. In China, MMA was first described in the year 2000 [4, 5]. Between 2002 and 2022, several studies have explored the incidence rate of MMA, which has been estimated as 1:15,200 [6]. The prevalence varies widely geographically: 1/26,000 in Beijing and Shanghai [4], 1/3920 in Shandong [7], 1/33,300 in Zhejiang [8], and 1/11,160 in Henan [9]. The *MMACHC* gene mutation is the most common [10]. In 2018, a large multi-center retrospective study reported the phenotypes, genotypes, treatment, and prevention of 1003 MMA Chinese patients [11]. Other works reported clinical and biochemical features [12, 13], preferred therapeutic methods [14], and summarized that early screening and diagnosis of MMA, employing computational tools such as artificial intelligence technology [15], and next-generation sequencing [16, 17] can reduce the false positives effectively and greatly improve efficiency. These studies showed that early diagnosis and treatment are crucial for the survival and well-being of affected children. In Thailand, MMA cases have been reported and investigated in recent years and several works have described novel mutations in individual patients [18–20]. A larger-scale study in 2012 [21] reported the genotypes and phenotypes of Thai patients with MMA that were identified between 1997 and 2011. In 2017, a 5-year retrospective study [22] reported clinical and laboratory findings and outcomes of classic organic acidurias including MMA in children in north-eastern Thailand and their outcomes. Other works have reported co-occurring morbidities such as juvenile gout [23] and unusual clinical presentations such as one mimicking diabetic ketoacidosis [24]. The studies suggest that earlier diagnostics of MMA have the potential to provide improved outcomes for Chinese and Thai pediatric patients.

MMA is characterized by high genetic heterogeneity and results from several genotypes arising from specific mutations causing the inherited form of the disease [25]. Most common mutations are in the genes *MUT* (mut type), *MMAA* (cbIA type), *MMADHC* (cblD type), *LMBRD1* (cblF type), *ABCD4* (cblJ type), and *HCFC1* (cblX type). Mutations in *MCEE* result in a milder phenotype. Further recent studies have revealed significant variations of genotype prevalence on a global scale [10, 26–31].

The routine screening of newborn infants aims to discover babies with inherent disorders shortly after birth, and often before clinical suspicion or presentation, thus enabling early treatment. Mass spectrometry-based testing, when performed with high sensitivity, returns a large number of false positives, i.e., the tests are operating with high sensitivity and while they robustly capture the affected newborns, the results are beset with a high number of false positives. The test-screened individuals are, thus, segmented into test screen positive and test screen negative. The test-screen-negative individuals usually undergo no further assessment. In contrast, test-screen-positive individuals are often recalled for further tests with higher specificity to confirm (true positives) or reject (false positives) the diagnosis, aiming to establish a final clinical decision and commencing treatment. The aim of this work was to develop a computational methodology with utility in reducing false-positive results for MMA-suspected patients.

Newborn screening using tandem mass spectrometry (MS/MS) has transformed the ability of clinicians to identify and provide early, lifesaving treatment to infants with such hereditary metabolic diseases and remarkable efforts of international collaborations have established objective definitions of many biomarkers applicable to certain rare diseases [32]. MMA suspicion and diagnosis often occur in the early development stage of the pediatric patient via a neonatal routine MS/MS screen or clinical tests upon presentation. Specifically, MMA is detected with a focus on levels of propionyl-carnitine (C3) and its ratio with acetyl-carnitine (C3/C2) [32]. While screening using tandem mass spectrometry (MS/MS) does detect most newborns with MMA, it also returns a high number of false-positive results (5 FP to 1 TP). This leads to a slew of further biochemical and genomic tests of all positives, to confirm or reject the initial result and arrive at a final diagnosis. This iterative strategy quite often results in multiple visits to the medical clinics, diagnostics delays, and unnecessary burdens on the health system, the clinicians, the medical facilities and laboratories, and most importantly on the patients and their families. Novel primary screening approaches and implementation of secondary-tier tools enabling a dramatic reduction of the false-positive results in newborn screening are necessary to reduce these undesirable effects [33].

While notable advances have taken place in the diagnosis and management of MMA, unanswered questions still remain. Thus, investigative studies that integrate available clinical screening datasets with advanced multivariable analysis methods can enhance the knowledge of MMA and result in improved screening and early diagnostics. The study presented herein focuses on the development of improved classification models with increased precision. The objective of this work is to address pressing unmet needs in the clinical management of MMA and utilize metabolite data to develop an improved classification methodology for MMA. The goal is to develop a secondary tier classification model with a focus on increased precision that is run downstream from any currently available classification test of MMA, which aims to discover the false positives in the upstream (prior) diagnostic classification. The results of these efforts have the potential to greatly benefit the diagnostics of this disease in children in Thailand, China, and globally.

## Methods

**Fig. 1:**
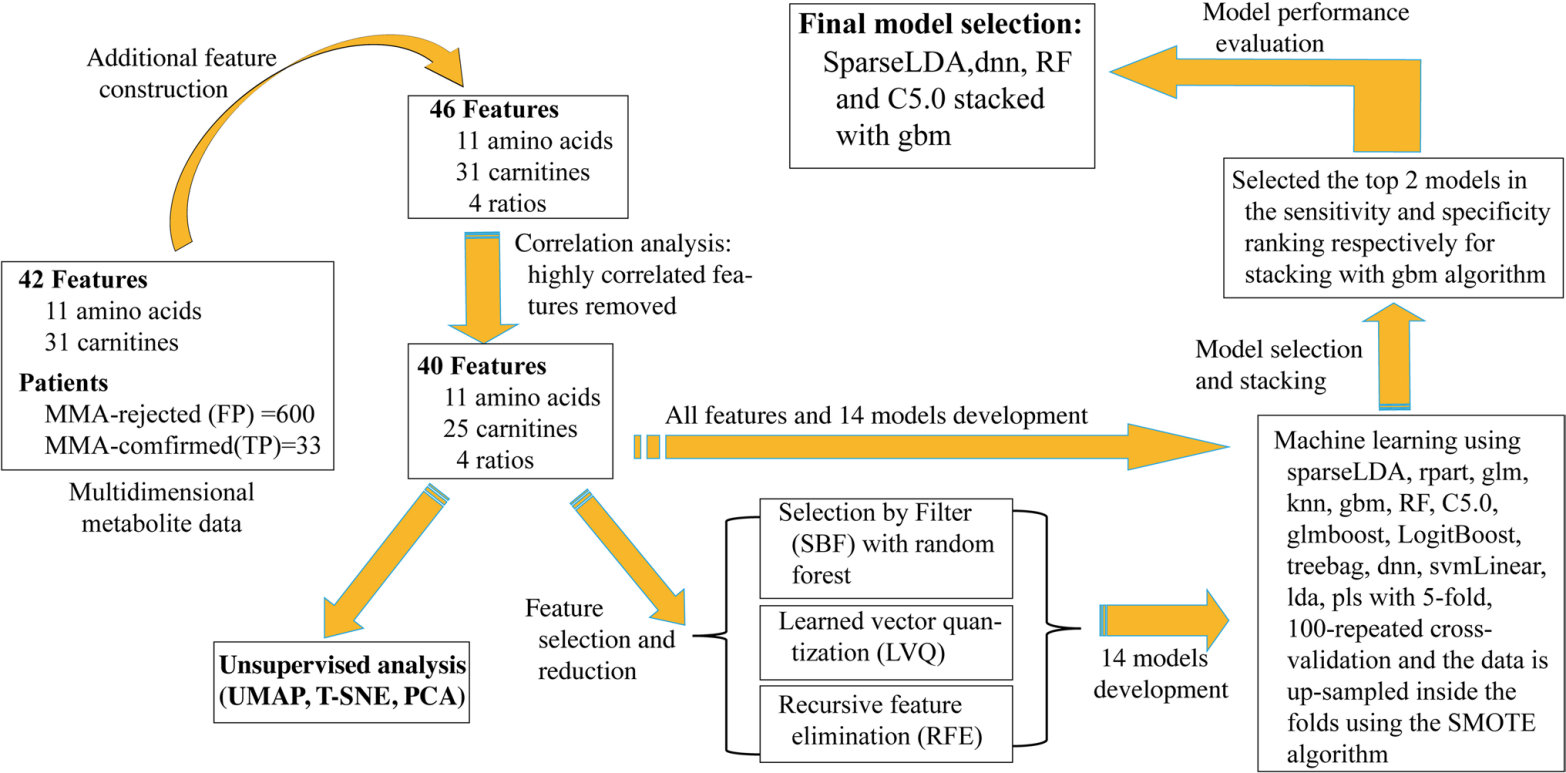
Process flowchart. *UMAP* uniform manifold approximation and projection, *t-SNE* t-distributed stochastic neighbor embedding, *PCA* principal component analyses, *sparseLDA* sparse linear discriminant analysis, *rpart* CART, *glm* generalized linear model, *knn* k-nearest neighbors, *gbm* stochastic gradient boosting, *RF* random forest, *glmboost* booted generalized linear model, *LogitBoost* boosted logistic regression, *treebag* bagged CART, *dnn* deep neural network, *svmLinear* support vector machines with linear kernel, *lda* linear discriminant analysis, *pls* partial least squares, SMOTE synthetic minority oversampling technique

To address the above-stated unmet need in the management of MMA, we utilized mass spectrometry neonatal data, feature selection strategies, and machine learning methods to develop multivariable screening models for MMA as a secondary tier tool for false positives reduction. The workflow (Fig. 1) consists of data processing, additional feature construction, feature selection and reduction, unsupervised analysis, model development, model performance evaluation, and final model selection.

### Study population

A real-world dataset of MMA screen-positive individuals was collected at the Newborn Screening Center of Shanghai Children’s Hospital. The data encompasses the time period between 2013 and 2019, for a total of 218,489 patient records, and consists of children who underwent routine newborn screening or came with a clinical referral. Patients fulfilling either one of the following two screening cut-offs: C3 > 5 μmol/L and C3/C2 > 0.25 μmol/L were deemed MMA-screen-positive. A total of  633 patients were included in the screen-positive dataset. Subsequent secondary diagnostic testing including combined secondary LC–MSMS tests, GC–MS tests, and gene tests were used to confirm or reject suspicion of MMA. The patient cases were, thus, labeled as MMA-screen-true positive-MMA confirmed true positive (TP) for 33 cases and MMA-screen-false positive-MMA-rejected false positive (FP) for  600 cases. To protect the privacy and anonymity of the patients, all patient-identifying information was removed and obfuscated before proceeding with the analytical treatment.

### Sample collection and mass spectrometry

Dried blood samples (DBS) are routinely collected at the Newborn Screening Center of the Shanghai Children’s Hospital. A tandem mass spectrometry system with a mass spectrometer (MSMS, Waters Quattro micro, Milford, Massachusetts, USA), high-performance liquid chromatography (Waters 1525 u Binary HPLC Pump), an automatic sampling system (Waters 2777 Sample manager), and a non-derivative tandem mass spectrometry kit (NeoBaseTM Non-derivatized MSMS Kit, PerkinElmer, Waltham, Massachusetts, USA) were utilized to measure the 11 amino acids and 31 carnitines. For each DBS, 3 mm diameter circles were punched into a 96-well microplate and 100 μl of the extraction solution containing methanol/water (80:20 v/v), stable isotope internal standard containing amino acid internal standard [^2^H_4_-alanine (^2^H_4_-ALA); ^2^H_4_,^13^C-arginase (^2^H_4_,^13^C-ARG); ^2^H_2_-citrulline (^2^H_2_-CIT); ^15^N,2-^13^C-glycine (^15^N,2-^13^C-GLY); ^2^H_3_-LEU; ^2^H_3_-methionine (^2^H_3_-MET); ^2^H_6_-ornithine (^2^H_6_-ORN); ^13^C_6_-phenylalanine (^13^C_6_-PHE); ^13^C_5_-proline (^13^C_5_-PRO), ^13^C_6_-tyrosine (^13^C_6_-TYR); ^2^H_8_-VAL)], and carnitines internal standard [^2^H_9_-carnitine (^2^H_9_-C0); ^2^H_3_-C2, ^2^H_3_-C3, ^2^H_3_-butyryl-carnitine (^2^H_3_-C4); ^2^H_9_-lsovaleryl-carnitine (^2^H_9_-C5); ^2^H_6_-glutaryl-carnitine (^2^H_6_-C5DC); ^2^H_3_-hexanoyl-carnitine (^2^H_3_-C6), ^2^H_3_-octanoyl-carnitine (^2^H_3_-C8), ^2^H_3_-decanoyl-carnitine (^2^H_3_-C10), ^2^H_3_-dodecanoyl-carnitine (^2^H_3_-C12), ^2^H_3_-tetradecanoyl-carnitine (^2^H_3_-C14), ^2^H_3_-hexadecanoyl-carnitine (^2^H_3_-C16), ^2^H_3_-octadecanoyl-carnitine (^2^H_3_-C18)] was added, then the sample was incubated and oscillated at 45 °C and 650–750 r/minute for 45 minutes. After elution, 75 μl extraction of each sample was transferred into the V-shaped bottom detection plate, the plate was placed with a sleeve into the automatic sampler, and the program was started. The mobile phase was 84% acetonitrile, 16% water, and 0.1% formic acid, and the flow rate of the quaternary pump was set at variable speeds as follows: 0.11 mL/min from 0 to 0.10 minutes, 0.01 mL/min from 0.11 to 1.10 minutes, 0.8 mL/min from 1.11 to 1.60 minutes, and 0.11 mL/min from 1.61 to 2.00 minutes. Each sample had an injection volume of 20 μL and took 2 minutes for analysis.

### Additional feature construction, correlation analysis, and Wilcoxon test

The measured features included amino acids (*n* = 11) and carnitines (*n* = 31). Additional features were constructed from the metabolites: free carnitine (C0), acetyl-carnitine (C2), propionyl-carnitine (C3), hexadecanoyl-carnitine (C16), and MET. The feature construction step was performed by computing the ratios C3/C2, C3/C0, C3/C16, and C3/MET. A total of 46 features were used at the start of the downstream computational analysis. The features were scaled and centered [34]. Normalized values were used to perform the Pearson correlation analysis, which aims to find a linear relationship between the metabolites in order to identify highly correlated features. The correlation analysis was performed with the *stats::cor* function. The pairwise correlation matrix was computed using the *caret::findCorrelation* function. Features with a correlation of over 0.89 with another feature were removed. The removed features were 3-Hydroxy-octadecenoyl-carnitine (C18:1OH), 3-Hydroxy-hexadecanoyl-carnitine (C16OH), Tetradecenoyl-carnitine (C14:1), Dodecenoyl-carnitine (C12:1), 3-Hydroxy-octadecanoyl-carnitine (C18OH), and 3-Hydroxy-tetradecanoyl-carnitine (C14OH).

Next, we used the Wilcoxon test [35] to help identify the features that differed between screen-TP-MMA-confirmed (TP) and screen-FP-MMA-rejected cases (FP). Split violin plots were utilized to visualize the concentration differences between groups. The R function *stat::wilcox.test()* was used to perform the Wilcoxon test.

### Unsupervised analysis by uniform manifold approximation and projection, *t*-distributed stochastic neighbor embedding, and principal component analyses

Using the patient dataset consisting of  40 features (after the 6 highly correlated features were removed), t-distributed stochastic neighbor embedding (t-SNE), uniform manifold approximation and projection (UMAP), and principal component analyses (PCA) were operated utilizing the appropriate function from the *Rtsne*, *umap*, and *mixOmics* R packages, namely *Rtsne::Rtsne, umap::umap, and mixOmics::pca*. For the t-SNE, perplexity was set to number 50, and 1000 iterations were performed. The number of neighbors in UMAP was 15.

### Feature selection for parsimonious modeling

We applied three feature selection methods: selection by filter (SBF) with the random forest (RF) algorithm, learned vector quantization (LVQ) [36, 37], and recursive feature elimination (RFE) with the RF algorithm. The goal of utilizing multiple methods was to check for consistency of the selected feature set and to aim for consensus discovery and diversity for machine learning model input. LVQ was performed with the *caret* R package [38] using the functions *caret::trainControl and caret::train* with parameter model = “lvq”. After the model was fitted, features were ranked by importance using the *caret::varImp* function. Cohen’s Kappa and accuracy were utilized as performance metrics. RFE with the RF algorithm was performed using *caret::rfeControl*, *caret::trainControl*, and *caret::rfe*. SBF with the RF algorithm was performed using the functions *caret::sbfControl* and *caret::sbf*. Target performance metrics were sensitivity, specificity, and area under the receiver operating characteristic curve (AUROC). For the feature selection approaches, fivefold cross-validation repeated 100 times was performed and the data were up-sampled inside the cross-validation folds using the Synthetic Minority Oversampling Technique (SMOTE) algorithm [39] to address the class imbalance problem present in this MMA dataset.

### Algorithm selection, evaluation, and ensemble modeling by stacking

To discover a well-performing secondary-tier predictive model for the MMA,  14 models were fitted and evaluated. The following advanced machine learning classifiers were fitted utilizing the appropriate function in the R caret package: sparse linear discriminant analysis (sparseLDA), CART (rpart), generalized linear model (glm), k-nearest neighbors (knn), RF, C5.0, stochastic gradient boosting (gbm), boosted generalized linear model (glmboost), boosted logistic regression (LogitBoost), bagged CART (treebag), stacked autoencoder deep neural network (dnn), support vector machines with linear kernel (svmLinear), linear discriminant analysis (lda), and partial least squares (pls). The set encompassed a diverse set of linear, non-linear, and boosting methods. The full set of features (*n* = 40) and the features selected by the three procedures were employed for a total of  56 models fitted. Target performance metrics were sensitivity and specificity; fivefold cross-validation was repeated 100 times and the data were up-sampled inside the folds using the SMOTE algorithm [39] to address the class imbalance problem. The models were ranked by performance measured by sensitivity and specificity. The top two models in the sensitivity ranking and the top two models in the specificity ranking were selected for input into the stacking step. Based on the results of this step, a classification ensemble model for MMA consisting of four base models stacked with the gbm algorithm as the supervisor algorithm was developed as the final model. The *caret::CaretStack and caret:CaretList* functions in the caret *R* package were utilized.

### Final model validation and performance comparison

The results were subjected to fivefold cross-validation repeated 100 times to evaluate the classification model and ensure that the final model achieved a satisfactory performance in the classification task. Commonly utilized metrics such as sensitivity, specificity, false-positive rate (FPR), and area under the curve (AUC) were computed as measurements to evaluate the discriminatory power of the final classification model.

## Results

### Overview of the data, correlation analysis, and Wilcoxon test

The metabolite dataset in the study was first summarized with highly correlated features removed, and then explored via unsupervised analysis. Median and range values for each metabolite were computed. A boxplot of the scaled and centered data, including 46 features, is shown in Supplementary Fig. S1. Analytical methods were then applied to the data to filter out highly correlated and irrelevant features, avoid overfitting, select the optimal feature signature for the MMA screening, and develop an optimized model. The correlation between features was assessed by Pearson correlation analysis and the highly correlated features were targeted for removal. Utilizing a convenient cutoff of correlation coefficient = 0.89, six features (C18:1OH, C16OH, C14:1, C12:1, C18OH, and C14OH) were found to be highly correlated to another feature in the dataset. Thus, these features were removed from the dataset for the downstream analysis to arrive at  40 features. The correlation matrix is presented in Supplementary Fig. S2. Next, we compared the two groups using the Wilcoxon test which assesses whether the mean values for each metabolite differ significantly from each other for the two classes. The results of the Wilcoxon test are depicted as violin plots in Supplementary Fig. S3 and with extended details in Table 1.

**Table 1:** Metabolites concentration levels and Wilcoxon test statistic comparing MMA-rejected (FP) and MMA-confirmed (TP) groups

Metabolites	FP group (median)	TP group (median)	W	*P* value
ALA	322.19	271.39	13,185	0.001
ARG	12.17	12.51	8356.5	0.131
CIT	15.24	17.89	8648	0.221
GLY	375.95	259.94	13,388	0.001
LEU	166.73	150.99	11,194	0.206
MET	22.25	9.51	16,578	< 0.001
ORN	101.53	83.93	12,696	0.006
PHE	53.14	46.40	12,364	0.016
PRO	184.80	153.87	12,484	0.012
TYR	80.88	57.55	13,057	0.002
VAL	143.03	137.98	10,371	0.646
C0	31.64	12.91	15,184	< 0.001
C2	25.08	13.55	12,605	0.008
C3	5.11	7.71	3897.5	< 0.001
C3DC + C4OH	0.13	0.12	10,704	0.431
C4	0.26	0.20	12,043	0.036
C4DC + C5OH	0.24	0.21	10,230	0.747
C5	0.15	0.17	9762	0.893
C5:1	0.02	0.02	7183	0.004
C5DC + C6OH	0.14	0.12	11,582	0.100
C6	0.05	0.05	10,128	0.823
C6DC	0.10	0.11	9708.5	0.851
C8	0.06	0.05	10,310	0.687
C8:1	0.14	0.15	9074.5	0.419
C10	0.06	0.06	10,152	0.805
C10:1	0.08	0.09	9029	0.393
C10:2	0.02	0.02	10,413	0.591
C12	0.06	0.05	11,354	0.154
C14	0.17	0.08	14,720	< 0.001
C14:2	0.02	0.02	9573.5	0.738
C16	1.94	0.80	15,018	< 0.001
C16:1	0.11	0.05	14,390	< 0.001
C16:1OH	0.03	0.03	9204	0.488
C18	0.66	0.41	14,914	< 0.001
C18:1	1.31	0.80	14,118	< 0.001
C18:2	0.32	0.26	11,847	0.057
C3/C0	0.13	0.58	1104.5	< 0.001
C3/C2	0.24	0.73	1096	< 0.001
C3/MET	0.17	0.80	1138	< 0.001
C16/C3	0.47	0.10	17,779	< 0.001

*ALA* Alanine, *ARG* Arginine, *CIT* Citrulline, *GLY* Glycine, *LEU* Leucine, *MET* Methionine, *ORN* Ornithine, *PHE* Phenylalanine, *PRO* Proline, *TYR* Tyrosine, *VAL* Valine, *C0* free-carnitine, *C2* acetyl-carnitine, *C3* propionyl-carnitine, *C3DC + C4OH* malonyl-carnitine + 3-hydroxybutyryl-carnitine, C4 butyryl-carnitine, *C4DC + C5OH* methylmalonyl-carnitine + 3-hydroxyisovaleryl-carnitine, C5 ﻿lsovaleryl-carnitine*, C5:1* tiglyl-carnitine, *C5DC + C6OH* glutaryl-carnitine + 3-hydroxyhexanoyl-carnitine, C6 hexanoyl-carnitine, *C6DC* methylglutaryl-carnitine, *C8* octanoyl-carnitine, *C8:1* octenoyl-L-carnitine, *C10* ﻿decanoyl-carnitine, *C10:1* ﻿decenoyl-carnitine, *C10:2* ﻿decadienoyl-carnitine, *C12* ﻿dodecanoyl-carnitine, *C14* ﻿tetradecanoyl-carnitine, *C14:2* ﻿tetradecadienoyl-carnitine, *C16* hexadecanoyl-carnitine, *C16:1* hexadecenoyl-carnitine, *C16:1OH* 3-hydroxy-hexadecenoyl-carnitine, *C18* octadecanoyl-carnitine, *C18:1* octadecenoyl-carnitine, *C18:2* octadecadienoyl-carnitine

### Unsupervised analysis by uniform manifold approximation and projection, *t*-distributed stochastic neighbor embedding, and principal component analyses

**Fig. 2:**
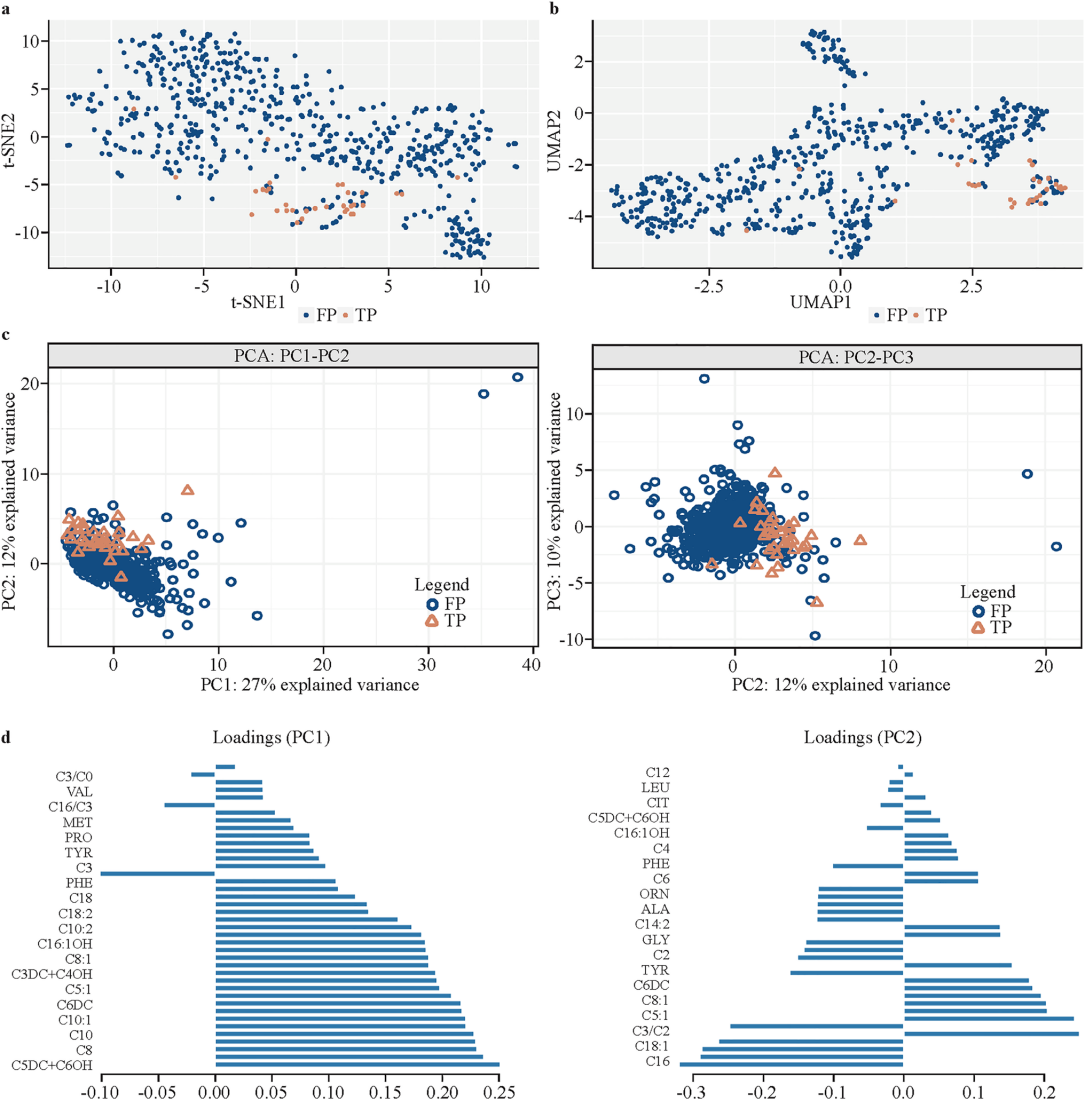
Unsupervised analysis results of t-SNE, UMAP, and PCA. **a** t-SNE visualization of the *n* = 40 analytes data. Class screen-TP/MMA-confirmed is shown as pink dots and class screen-FP/MMA-rejected is shown as blue dots. **b** UMAP visualization of the *n* = 40 analytes data. Class screen-TP/MMA-confirmed is shown as pink dots and class screen-FP/MMA-rejected is shown as blue dots. **c** PCA score plots for the PC1, PC2, and PC3. **d** Loading plots. *UMAP* uniform manifold approximation and projection, *t-SNE* t-distributed stochastic neighbor embedding, *PCA* principal component analyses, *TP* true positive, *FP* false positive, *MMA* methylmalonic acidemia, *PC* principal component, C0 free carnitine, C2 acetyl-carnitine, C16 hexadecanoyl-carnitine, VAL valine, MET methionine, PRO proline, TYR tyrosine, C3 propionyl-carnitine, PHE phenylalanine, C18 octadecanoyl-carnitine, C18:2 octadecadienoyl-carnitine, C10:2 decadienoyl-carnitine, C16:1OH 3-hydroxy-hexadecenoyl-carnitine, C8:1 octenoyl-L-carnitine, C3DC + C4OH malonyl-carnitine + 3-hydroxybutyryl-carnitine, C5:1 tiglyl-carnitine, C6DC methylglutaryl-carnitine, C10:1 decenoyl-carnitine, C10 decanoyl-*carnitine, C8 octanoyl-carnitine, C5DC + C6OH glutaryl-carnitine + 3-hydroxyhexanoyl-carnitine, LEU leucine, CIT citrulline, C4 butyryl-carnitine, PHE phenylalanine, C6* *hexanoyl-carnitine, ORN ornithine, ALA alanine, C14:2 tetradecadienoyl-carnitine, GLY glycine, C2 acetyl-carnitine, TYR tyrosine, C18:1 octadecenoyl-carnitine, C16* hexadecanoyl-carnitine

The unsupervised methods t-SNE, UMAP, and PCA were carried out on the data. T-SNE was utilized to calculate a visualization of the structure of the data. The t-SNE-derived visualization of the 40 features revealed distinct group-specific separation (Fig. 2a). The figure shows the MMA-confirmed samples (red dots, *n* = 33) are clustered together and yet interspersed and enclosed within MMA-rejected samples class space (blue dots,* n* = 600).

UMAP analysis was performed on the data before and after the correlation analysis. UMAP is a method utilized as a non-linear dimensionality reduction scheme as well as to find intrinsic structure and cluster members of a dataset; it can also be used to analyze different types of data. When performing UMAP analysis, a weighted *k*-nearest neighbor graph is first calculated, and then a layout in lower dimension is created. After that, the data can be graphed, visualized, and its global structure examined in the reduced-dimensionality space. Similar to the t-SNE results, UMAP shows clustering and enclosure of the positive class within the negative class space (Fig. 2b).

PCA achieved the following explained variance per component (PC1, PC2, and PC3): 27%, 12%, and 10% for a cumulative explained variance of 49% (Fig. 2c and d). Similar to t-SNE and UMAP, PCA shows clustering and enclosure of the positive class within the negative class space, thus more advanced machine learning methods can be utilized. Overall, the unsupervised results emphasize the need to employ supervised classification methods with further sophistication, suggesting that machine learning techniques could be employed to separate the classes.

### Feature selection

For feature selection, we used automatic feature selection methods that proceed to build a set of models and identify the features important in building an accurate classification model. The method we applied was SBF, which involved model fitting after applying univariable filters. This method is able to pre-screen the predictors using simple univariable statistical methods, but only use those that pass some criterion in the subsequent model steps. The following features were selected: ALA, GLY, MET, ORN, PHE, PRO, C0, C2, C3, C14, C16, hexadecenoyl-carnitine (C16:1), 3-hydroxy-hexadecenoyl-carnitine (C16:1OH), C18, octadecenoyl-carnitine (C18:1), C3/C0, C3/C2, C16/C3, and C3/MET. This was the feature set that resulted in the best model classification performance in the downstream modeling step.

For comparison, we also applied LVQ [36] and RFE with the RF algorithm. LVQ can be utilized to rank feature importance in generating a predictive model. This, and the related method of self-organizing maps, were developed by Kohonen et al. [37]. Using the *varImp* function in the *caret* package, we computed feature importance ranking for the 40 metabolites. A fivefold cross-validation was repeated 100 times. The most important features were ranked as C3/C2, C3/C0, C3/MET, C16/C3, MET, and C3, which is in concordance with the use of these features in clinical practice. Supplementary Fig. S4a shows the rankings of all 40 features.

Another automated method that we utilized was RFE, wherein a subset of features can be explored and the RF algorithm evaluates the model. All 40 features were selected in our analysis. The variables vs. ROC plot (Supplementary Fig. S4b) shows that using *n* = 18 variables achieves slightly better results. For the RFE algorithm, the selected features were CIT, GLY, MET, VAL, C0, C2, C3, malonyl-carnitine (C3DC) + 3-hydroxybutyryl-carnitine (C4OH), C5, C5DC + 3-hydroxyhexanoyl-carnitine (C6OH), C14, C16:1, C16:1OH, C18, C3/C0, C3/C2, C16/C3, and C3/MET.

### Classification modeling by evaluating algorithms and stacking

**Fig. 3:**
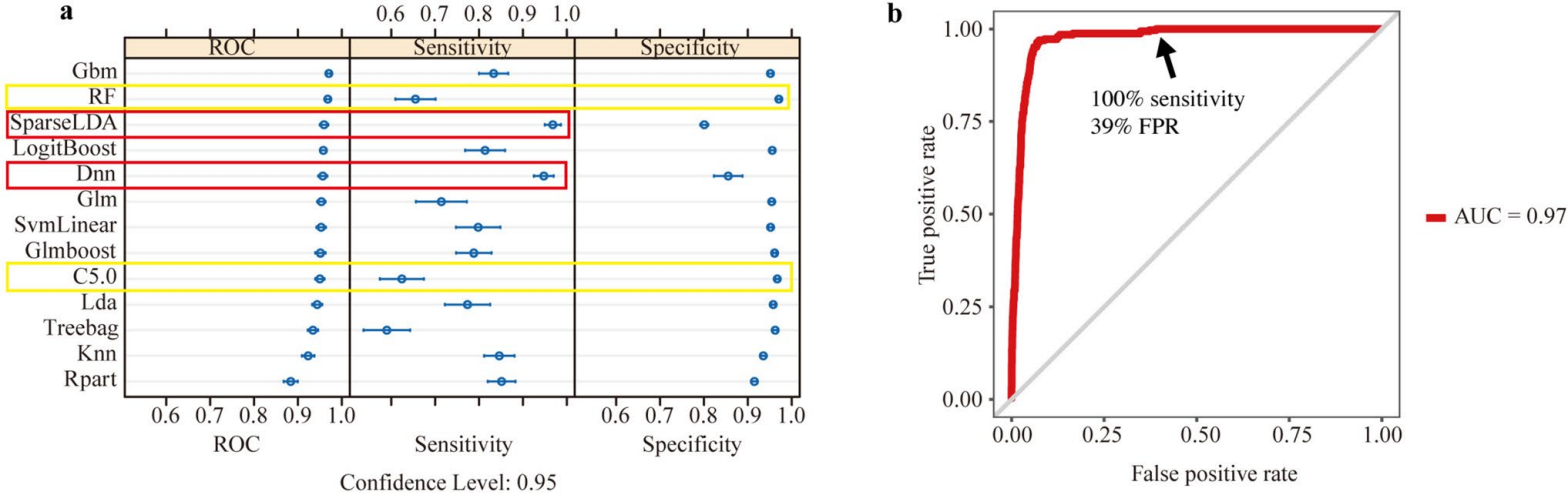
Model assessments and stacking ensemble result. **a** Model assessments of ROC, sensitivity, and specificity of the 14 models to classify screen-positive MMA patients. **b** Stacking ensemble result included the algorithms RF, C5.0, sparse linear discriminant analysis, and autoencoder deep neural network stacked with the algorithm stochastic gradient boosting as the supervisor, using AUROC and FPR at 100% sensitivity. Results were obtained via a fivefold, 100 times repeated cross-validation utilizing the SBF feature selection derived feature set. ROC receiver operating characteristic curve, AUC area under the curve, MMA methylmalonic acidemia, AUROC area under the receiver operating characteristic curve, *gbm* stochastic gradient boosting, *RF* random forest, *glmboost* booted generalized linear model, *LogitBoost* boosted logistic regression, *treebag* bagged CART, *dnn* deep neural network, *svmLinear* support vector machines with linear kernel, *lda* linear discriminant analysis, *sparseLDA* sparse linear discriminant analysis, *rpart* CART, *glm* generalized linear model, *knn* k-nearest neighbors

We explored the performance of 14 classification models (Fig. 3a) utilizing the SBF feature selection features subset to identify candidate models for the stacking step. Results were obtained via a fivefold cross-validation, repeated 100 times. Most models achieved similar specificity, except for sparseLDA. The sensitivity results ranged from about 60% to 90% sensitivity, except for sparseLDA*,* which achieved a sensitivity of over 95%.

The models were ranked by sensitivity and specificity and a set of four models combining the top two models in each category (RF and C5.0 for specificity, and sparseLDA and dnn for sensitivity) were stacked with gbm as the supervisor algorithm. Before the stacking, we confirmed that the predictions of the sub-model were not highly correlated (correlation coefficient > 0.89) as in this case; they would be making the same or very similar predictions, thus reducing the benefit of combining the predictions via the stacking approach. The additional three feature selection schemes: no feature selection, RFE-ROC, and LVQ were evaluated (Supplementary Fig. S5a, c, and e). The top-performing models in each of the three additional feature selection schemes are shown in Supplementary Table S1.

The best result was achieved using RF, C5.0, sparseLDA, and dnn, stacked with gbm as the supervisor. Evaluated in fivefold cross-validation repeated 100 times, the stacking approach achieved an AUROC of 97%, a sensitivity of 92%, and a specificity of 95%. When sensitivity was set to 95%, 99%, and 100%, the models achieved an FPR of 6%, 35%, and 39% respectively (Fig. 3b).

In comparison, we performed the stacking utilizing an additional three input feature sets: all the 40 features, the features selected by LVQ, and the features selected by RFE. These input sets resulted in inferior classification outcomes (Supplementary Fig. S5b, d, and f).

## Discussion

In this work, we performed unsupervised and supervised statistical modeling and machine learning model building in order to discern a classification approach to reduce false positives in the first-tier MMA screen test which utilizes clinical cut-offs. Current screening tests that interrogate mass spectrometry data of newborn children together with clinically defined cut-offs can capture the vast majority of MMA-inflicted individuals; however, a large number of false positives (TP:FP = 1:5) are returned. Our work identifies computational models that explore additional metabolic analytes in order to reduce the number of false positives without compromising sensitivity. The model achieved an FPR of 39% at 100% sensitivity.

One particular aspect to consider when developing and optimizing the model is the selection of which features to include. While recent works have shown the utility of the inclusion of a wider range of features, a highly parsimonious model has the potential for increased clinical interpretability and reduced overfitting. A mass spectrometry-derived dataset can contain features that are highly correlated to each other and quite often it is preferable to remove the highly correlated features to achieve improved model performance. To account for this, we performed correlation analysis and computed the pairwise correlation coefficient for each of the 46 available features which led to the removal of six metabolic analytes from the downstream modeling steps due to their high correlation with another feature in the dataset. We used three automated feature selection strategies—LVQ, RFE with RF, and SBF with RF. These methods fit a set of models and identify the features important in building a well-performing classification model. The methods are also effective in selecting features in the input dataset that have the highest relevance in predicting the target class. LVQ is a self-organizing neural network method that can be used to rank the importance of the input features and to select a parsimonious feature set in downstream modeling. RFE with RF is another feature selection strategy that is well suited in the context of binary classification that was utilized to select a comparative set of candidate features, while at the same time optimizing a classification model. Finally, SBF performs model fitting after applying univariable filters which aim to pre-screen the predictors. Only predictors that pass some criterion are utilized in the subsequent model steps. The goal of utilizing several methods was to check for the consistency of the selected feature set and to achieve an optimal input set into the machine learning step. The resulting reduced feature sets were used as inputs into the stacked model with the SBF-selected feature set resulting in optimal performance. While works have shown that including a wide set of predictors benefits classification performance, our results also point to the fact that adding more features is unlikely to continue improving classification performance with metabolomics data and instead the utilization of a targeted panel may be preferable. The aim of utilizing the three methods was to check for consistency of the selected feature set and achieve an ensemble effect.

Further to this, we wanted to build the secondary prediction model to reduce the number of false positives without introducing many false negatives. The goal was to tune a statistical approach that significantly reduces the number of false-positive MMA cases (screen positives that turn out to have the diagnosis rejected). We explored the performance of 14 classification models. While most models achieved high specificity and passable sensitivity, sparseLDA achieved high sensitivity but suffered from low specificity, unsurprisingly due to the utilization of the clinical features that are employed in the cut-off-based clinical first-tier classification.

A suitable approach to improve the classification performance of the MMA dataset is to combine the prediction of several models in an ensemble prediction approach. In this work, we utilized stacking which involved building multiple models ideally of different types, and a supervisor model which learns to optimally combine the prediction of the sub-models. When the predictions of the sub-models are combined with stacking, the prediction sets of the sub-models should be ideally of low correlation. This fact suggests that the models perform and optimize their prediction sets not in the same but rather in different ways; thus allowing the supervisor classifier to summarize and achieve an improved score. If the predictions for the sub-models were highly correlated (correlation coefficient > 0.89) then they would be making the same or very similar predictions, most of the time reducing the benefit of combining the predictions.

The stacked machine learning algorithm was able to capture 39% of false positives in the screen-positive results which still fell short of a specificity and sensitivity of 100%, the goal of this work. Research in this direction is ongoing, with example works of the recent past including the development of a biomarker identifier algorithm powered by logistic regression analysis [40] and other research from the same group [41–43].

Our approach combining a four-model ensemble and a supervisor algorithm (gbm) in a stacking scheme achieved an FPR of 39%. In recent work [44], an approach utilizing RF operated on a dataset of MS/MS newborn screening analytes reduced the number of false positives by approximately half, while maintaining the original sensitivity (96.1%) of the primary diagnostic test and nearly double the positive predictive value (PPV; 16.5%–28.9%). Further work [45] by the group utilized 39 metabolic analyte measurements and clinical features from screen-positive patients reported in the California DBS program. The fitted RF model was able to reduce the number of FP in MMA by 45% (and in a few other metabolic diseases as well). In 2023, Mak et al. [46] expanded the metabolite panel to be utilized in the second-tier testing of DBS samples and fitted an RF on screen-positive cases sourced by the California NBS program, consisting of true- and false-positive in MMA and other rare metabolic diseases. This expanded approach, while maintaining 100% sensitivity, reduced false positives for MMA by 84%, achieving a ROC of 0.98 and PPV of 59.1%.

The results of this study are comparable with the most current state-of-the-art of secondary-tier classification albeit utilizing a more parsimonious dataset (*n* = 40 in the current study vs. *n* = 121 in Mak et al.). The findings of Peng et al. [44, 45] and Mak et al. [46], emphasize the utility and potential clinical application of secondary machine learning tests to reduce the number of false alarms (false positives). Our expanded application of feature selection methods as well as advanced stacking approaches which combine the performance of an array of first-step predictors further emphasize the importance of both feature selection and model building the second-tier classification of MMA. A limitation of this work is that it is a retrospective study with the data obtained from one hospital setting. Thus, additional features (such as gestational weight and age) that can potentially further inform and strengthen the model are not consistently available for the patient dataset.

The classification results and approach of this research can be utilized by clinicians globally, to improve the overall discovery of MMA in pediatric patients. First, it may allow for increased precision in the interrogation of results of the neonatal metabolomics screen. Specifically, it offers an improved classification methodology with direct applicability in clinical diagnostics of the disease. This classification model has improved precision and reduced Type I errors (reduced false positives), which is the situation when a healthy proband is labeled as having the disease. The model pinpoints the false positives within the results of already performed diagnostic tests. This helps determine if the positive result of a patient’s MMA neonatal screening is incorrect. The improved method, when adjusted to 100% precision, can be utilized to further inform the diagnostic journey of MMA, which may help to reduce the emotional and economic burden in the process of diagnosis of MMA for Thai and Chinese patients and their families, and the load on the public health system.

In conclusion, we analyzed metabolite profiles from neonatal screenings and performed unsupervised and supervised statistical modeling intending to develop stacked ensemble machine learning models with the potential to reduce false positives in primary MMA diagnostic results. The explored metabolite features showed highly significant altered states and relevant classification information in patients that had the primary MMA diagnosis confirmed or rejected by secondary clinical tests. Three unsupervised models—UMAP, t-SNE, and PCA—showed that MMA-confirmed samples were clustered together and also enclosed within the MMA-rejected samples class space, which motivated the need to develop supervised classification methods with further sophistication to separate the classes. Features selection by SBF was used and the final ensemble model of sparseLDA, dnn, RF, and C5.0 stacked with gbm obtained the best achievement. Our study underscored the fact that machine learning models can potentially be utilized as an auxiliary screening method in order to reduce the number of false positives in MMA diagnostics without compromising sensitivity.

## Supplementary Information

Below is the link to the electronic supplementary material.

Supplementary file1 (DOCX 6611 KB)

## Data Availability

The datasets generated during and/or analyzed during the current study are not publicly available due to involving human patient privacy and ethical restrictions. The data that support the findings of this study are available from the corresponding author on reasonable request.
