# Supplementary material for: Improving the second-tier classification of methylmalonic acidemia patients using a machine learning ensemble method
Source: World J Pediatr. 2024 Feb 24;20(10):1090–101. doi: 10.1007/s12519-023-00788-6 (PMC11502559; doi:10.1007/s12519-023-00788-6)
Supplement: Supplementary file 1 — Supplementary file1 (DOCX 6611 KB) [file 12519_2023_788_MOESM1_ESM.docx]

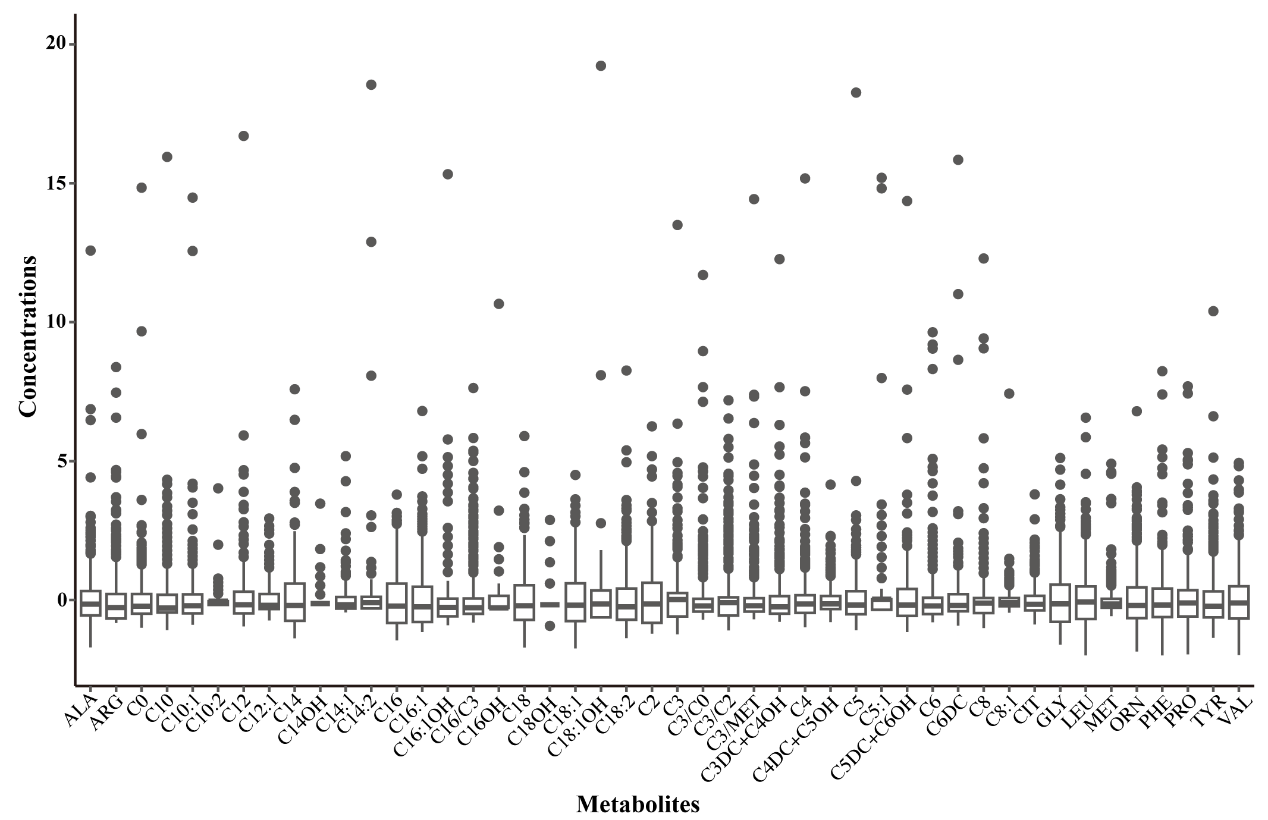


**Supplementary Fig. 1: Boxplot of the scaled and centered metabolite concentration data (n=46 features)**

**
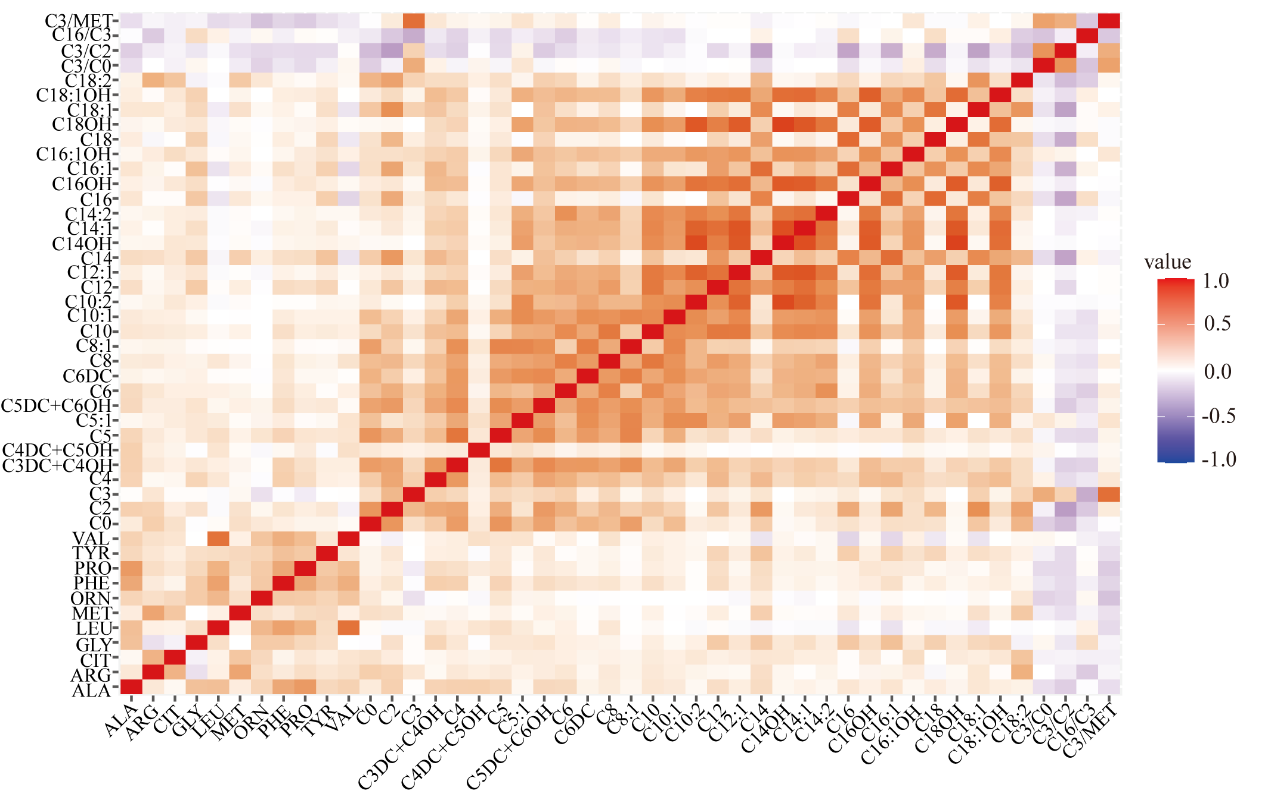
**

**Supplementary Fig. 2: Pair-wise Pearson correlation matrix of all n=46 features**

**
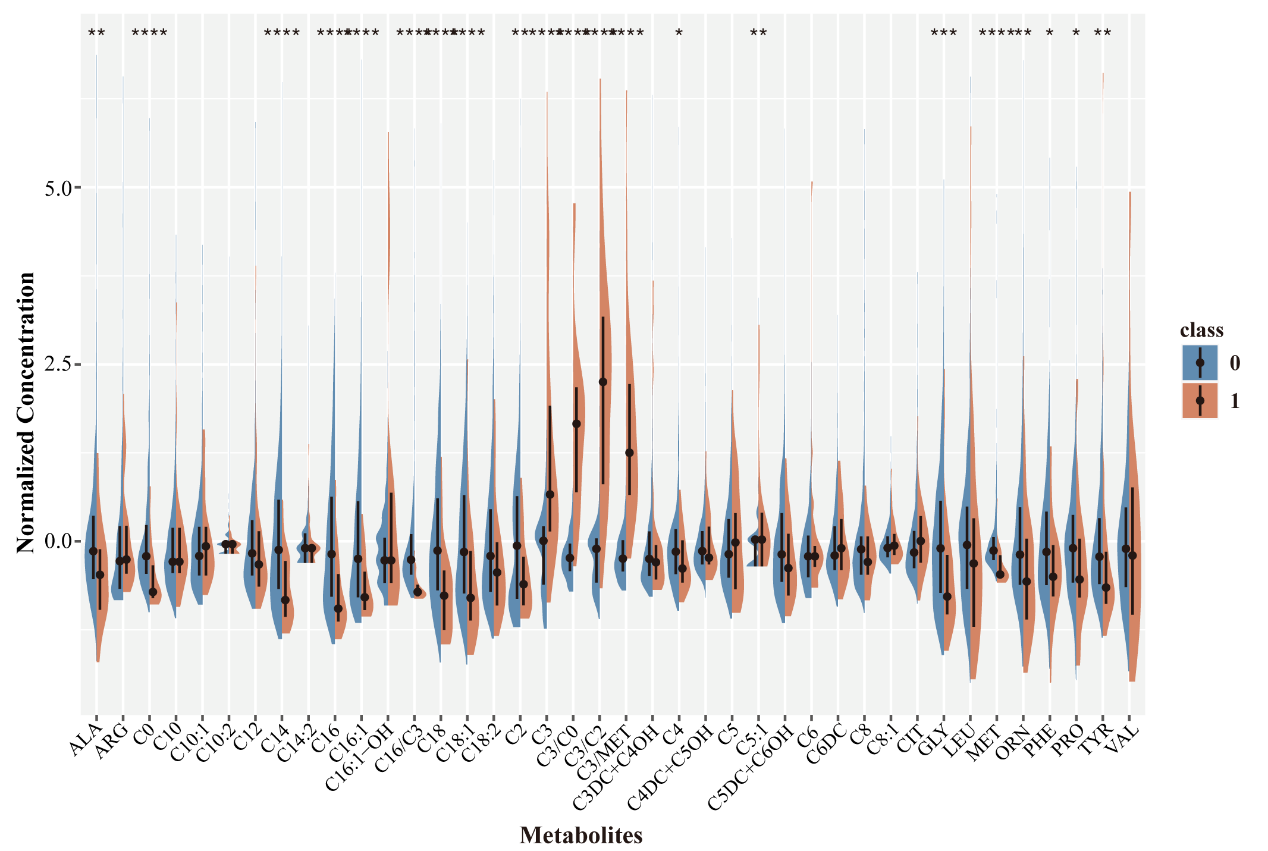
**

**Supplementary Fig. 3: Wilcoxon test result depicted by a violin plots.**

**The statistically significant mean differences between the 2 classes are labeled with an asterisk (*) above each violin plot. n=40 features are shown**

**
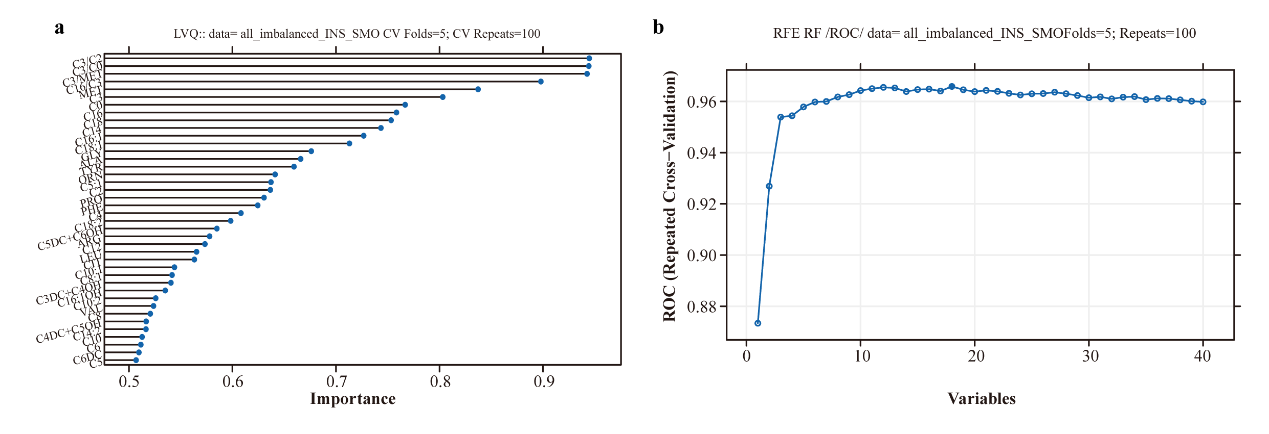
**

**Supplementary Fig. 4: (a) Feature importance ranking in utilizing the LVQ method. (b) RFE Feature selection with the RFE method operated on the dataset with n=40 features, the model is evaluated by random forest algorithm at each iteration**

**
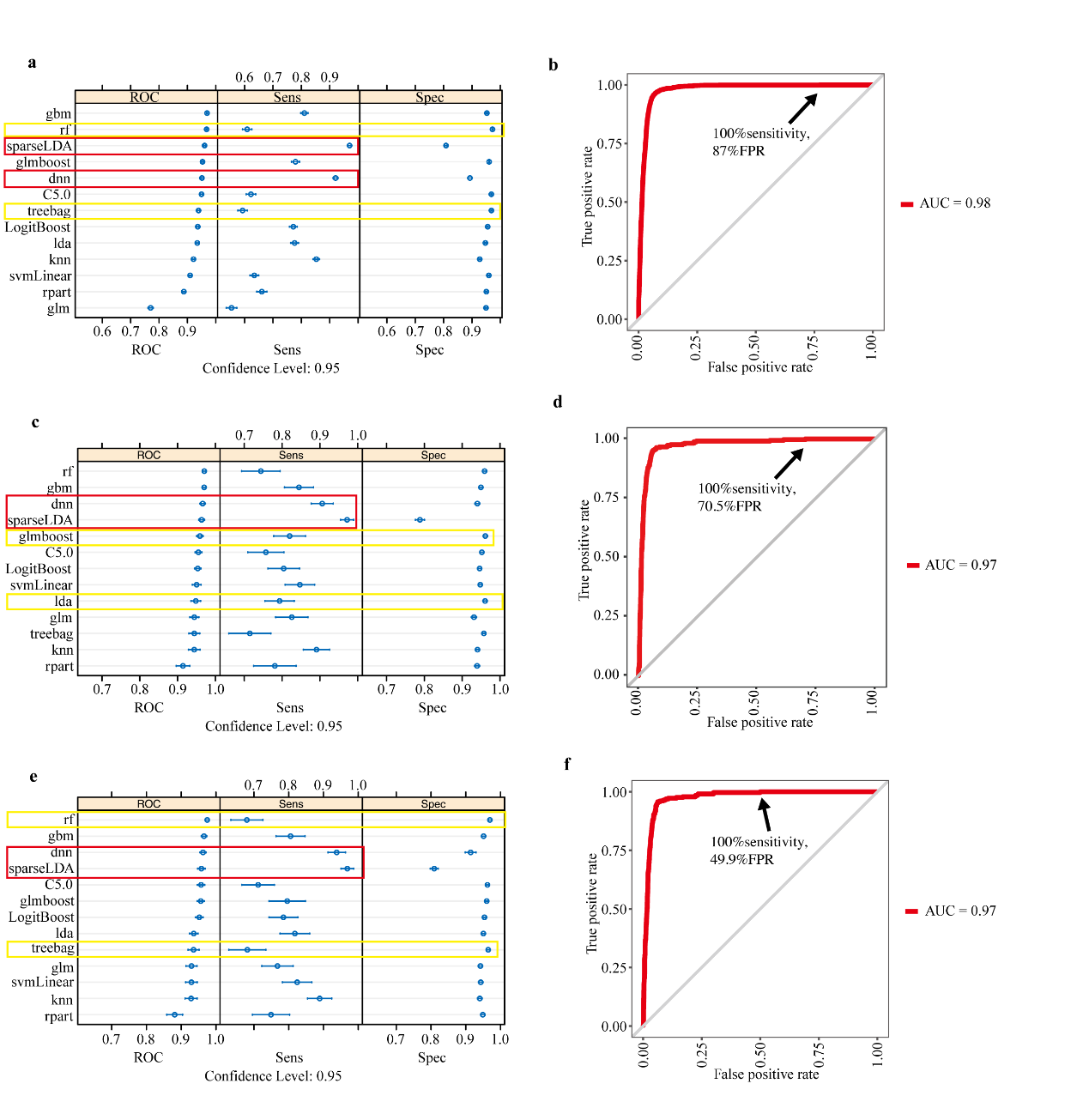
**

**Supplementary Fig. 5: Model assessments and stacking ensemble results.**

**(panel a, c, e) Model assessments of ROC, sensitivity and specificity of the 14 models to classify screen- positive MMA- patients. (panel b, d, f) AUROC, and FPR 100% sensitivity. Results obtained via a 5-fold, 100 times repeated cross validation utilizing the no feature selection (a, b), LVQ derived feature set(c, d), and RFE-ROC derived feature set (e, f). [1- Specificity = Probability that a true negative will test positive. = FP / N Also referred to as False Positive Rate (FPR) or False Positive Fraction (FPF)]**

**Supplementary Table I: Top 2 performing algorithms by specificity and sensitivity when no features selection and LVQ and RFE feature selection is employed**

| **Feature selection** | **Top 2 in specificity** | | **Top 2 in sensitivity** | |
| --- | --- | --- | --- | --- |
|  | **Model1** | **Model2** | **Model3** | **Model4** |
| ALL | *sparseLDA* | *dnn* | *rf* | *treebag* |
| LVQ | *sparseLDA* | *dnn* | *lda* | *glmboost* |
| RFE | *sparseLDA* | *dnn* | *rf* | *treebag* |
